# Optical microscopy approaches angstrom precision, in 3D!

**DOI:** 10.1038/s41377-019-0226-y

**Published:** 2019-12-11

**Authors:** Shi-Wei Chu

**Affiliations:** Department of Physics and Molecular Imaging Center, Taiwan University, https://www.ntu.edu.tw

**Keywords:** Super-resolution microscopy, Optical properties and devices

## Abstract

By coupling dye molecules with a graphene layer and localizing the molecules through quantification of fluorescence lifetime quenching, a novel imaging system offers unprecedented 1-nm resolution with angstrom precision in the axial dimension.

It is a long-standing goal to push the resolution of optical microscopy close to that of electron microscopy. In 2017, Nobel laureate Stefan Hell from Germany achieved a 1-nm resolution in the lateral dimension (xy plane) via MINFLUX^1^. Recently, Jörg Enderlein of the same country (also of the same town, Göttingen) has extended the same resolution to the third dimension^2^.

To enhance the spatial resolution, one well-known approach is to localize the emission from a molecule and separate individual molecules in a dimension that is orthogonal to space, such as wavelength, polarization, or time. For example, Christopher Cremer from Heidelberg University showed that fluorophores of different colors can be resolved with high precision^3^. Peng Xi from Peking University demonstrated that fluorescent molecules can be distinguished via dipole orientation mapping by polarization demodulation^4^. The localization imaging techniques developed by the 2014 Nobel laureates (William E. Moerner and Eric Betzig), as well as by Xiaowei Zhuang, distinguish fluorescent molecules by separating their emissions “stochastically” in time, whereas Stefan Hell’s method (STED) separates fluorophore emissions “deterministically” in time^5^.

With the premise that emission from a nearby fluorophore is separated, spatial resolution is given by the localization precision, which is determined by $L/\sqrt N$, where *L* is the interaction length and *N* is the number of photons. Therefore, significant resolution enhancement requires a small *L* or large *N*. The interaction length *L* can be reduced via a large gradient of the light intensity or of the light-matter coupling strength, and the number of photons *N* can be enhanced by using scattering or excitation laser photons directly. For instance, differential confocal microscopy achieves nanometer spatial resolution by placing a fluorophore at the edge of a tightly focused beam, i.e., reducing L^6^. Interferometric scattering microscopy reduces *L* via interference and increases N by localizing the scattering of a nanoparticle, the number of photons of which is typically much larger than that of a single fluorescent molecule^7^. MINFLUX enhances the spatial resolution by simultaneously reducing *L* and increasing N via the localization of a molecule with the steep intensity gradient of a focused donut laser beam.

Nevertheless, one major difficulty in all previous super-resolution techniques is realizing a similar resolution in the axial dimension (z) to that in the lateral dimensions (x and y) due to the asymmetric nature of focusing. A few years ago, Enderlein’s group demonstrated a few-nanometer resolution in the axial dimension by coupling dye molecules with a plasmonic film, i.e., metal-induced energy transfer (MIET)^8^. The ultrahigh axial resolution comes from detecting the quenching of the dye molecule fluorescence lifetime, which is also orthogonal to the spatial dimensions. The underlying physics is that fluorescence quenches when the dye molecules are close to the plasmonic film, resulting in a lifetime reduction that is primarily due to nonradiative energy transfer, i.e., coupling. The coupling strength is determined by the plasmonic evanescent field, which decays exponentially with an interaction length *L* ≈ 100 nm, thus creating a large gradient for high-precision localization.

The same group pushes the idea further by replacing the plasmonic film with a graphene film (g-MIET), which is a single atom in thickness^2^. The coupling between a dye molecule and graphene is similar to fluorescence energy transfer (FRET) between two molecules. That is, the coupling strength versus distance drops much more quickly than in a plasmonic film. The interaction length L is ~10 nm, one order smaller than that of plasmonics, and thus, the resolution of g-MIET can be much higher than that of MIET.

The principle of g-MIET is presented in Fig. 1. Similar to MIET, the axial position of the dye molecule is determined by the fluorescence lifetime variation. The closer the dye molecule is to the graphene film, the shorter the fluorescence lifetime (and the lower the photon yield). Apparently, the precision of the lifetime, as well as the precision of the axial position, depends on the number of photons *N*. When *N* reaches 10^7^, the precision attains an angstrom level, and the group successfully resolves not only dye molecules between two sides of a 5-nm-thick lipid bilayer membrane but also, impressively, the 1-nm gap between dye molecules and the substrate.

**Fig. 1:**
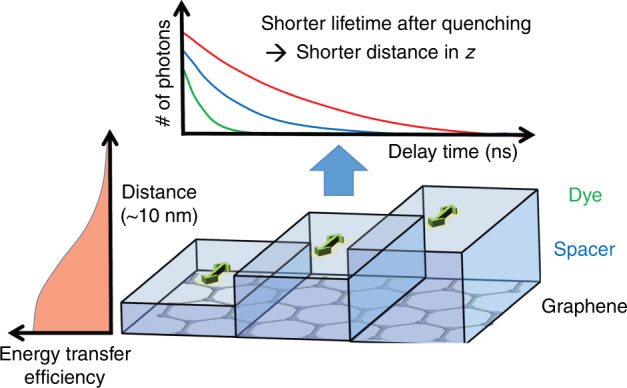
Principle of graphene-induced energy transfer for axial angstrom resolution. When dye molecules are close to a graphene layer, energy is transferred to the graphene, and the transfer efficiency strongly depends on the distance between them (left panel). The closer the molecules are to the graphene layer, the stronger the energy transfer and thus the shorter the fluorescence lifetime and the lower the photon yield (upper panel). When the lifetime variation is quantified with enough photons, the distance between dye molecules and the graphene layer is determined down to angstrom precision.

Compared to other super-resolution modalities, g-MIET provides unprecedentedly high axial resolution. If combined with other lateral resolution enhancing techniques, such as MINFLUX, optical imaging with isotropic 3D resolution approaching the subnanometer regime is anticipated. This resolution may bring the field of optical microscopy, or more appropriately “angstroscopy”, into the next epoch.

An interesting feature of g-MIET is that the coupling between graphene and the dye molecules works across the full visible spectrum and is not limited by the dye color. In addition, another potential advantage of g-MIET is the acquisition speed improvement, especially compared to that of conventional stochastic localization microscopies, for which the typical speed is less than one reconstructed frame per minute because the accumulation of a few thousand images is necessary. In the current scheme of g-MIET, the fluorescence lifetime is determined by time-correlated single-photon counting (TCSPC), which provides a 1–10 kHz pixel rate. With the recent development of high-bandwidth analog detection, it is possible to determine the fluorescence lifetime with each laser pulse and reach the MHz pixel rate in lifetime imaging, i.e., one 1024 × 1024 pixel image per second^9^.

It should be noted that the depth of g-MIET imaging is limited by the coupling length of graphene, i.e., ~10 nm, which is approximately the thickness of the plasma membrane. On the negative side, this limitation means that it is difficult to probe internal processes of a cell beyond the membrane. However, on the positive side, this limitation also helps to strongly constrain the observation region in the axial dimension, similar to total internal reflection microscopy, and could make g-MIET imaging an ideal tool for studying membrane biology.
